# Evaluation of two artificial infection methods of live ticks as tools for studying interactions between tick-borne viruses and their tick vectors

**DOI:** 10.1038/s41598-021-04498-9

**Published:** 2022-01-11

**Authors:** Camille Victoire Migné, Vaclav Hönig, Sarah Irène Bonnet, Martin Palus, Sabine Rakotobe, Clémence Galon, Aurélie Heckmann, Eva Vyletova, Elodie Devillers, Houssam Attoui, Daniel Ruzek, Sara Moutailler

**Affiliations:** 1 Anses, INRAE, Ecole Nationale Vétérinaire d’Alfort, UMR BIPAR, Laboratoire de Santé Animale, 94700 Maisons-Alfort, France; 2 INRAE, Anses, Ecole Nationale Vétérinaire d’Alfort, UMR VIROLOGIE, Laboratoire de Santé Animale, 94700 Maisons-Alfort, France; 3 Institute of Parasitology, Biology Centre, Czech Academy of Sciences, Branisovska 31, 370 05 Ceske Budejovice, Czech Republic; 4 Emerging Viral Diseases Research Group, Veterinary Research Institute, Hudcova 296/70, 621 00 Brno, Czech Republic; 5 Faculty of Agriculture and Faculty of Science, University of South Bohemia, Branisovska 31, 370 05 Ceske Budejovice, Czech Republic; 6 Present Address: Functional Genetics of Infectious Diseases Unit, Institut Pasteur, CNRS UMR 2000, Université de Paris, 75015 Paris, France

**Keywords:** Microbiology, Zoology

## Abstract

Up to 170 tick-borne viruses (TBVs) have been identified to date. However, there is a paucity of information regarding TBVs and their interaction with respective vectors, limiting the development of new effective and urgently needed control methods. To overcome this gap of knowledge, it is essential to reproduce transmission cycles under controlled laboratory conditions. In this study we assessed an artificial feeding system (AFS) and an immersion technique (IT) to infect *Ixodes ricinus* ticks with tick-borne encephalitis (TBE) and Kemerovo (KEM) virus, both known to be transmitted predominantly by ixodid ticks. Both methods permitted TBEV acquisition by ticks and we further confirmed virus trans-stadial transmission and onward transmission to a vertebrate host. However, only artificial feeding system allowed to demonstrate both acquisition by ticks and trans-stadial transmission for KEMV. Yet we did not observe transmission of KEMV to mice (IFNAR^−/−^ or BALB/c). Artificial infection methods of ticks are important tools to study tick-virus interactions. When optimally used under laboratory settings, they provide important insights into tick-borne virus transmission cycles.

## Introduction

Ticks represent significant risks for human and animal health. Because they are obligate hematophagous ectoparasites and feed on diverse vertebrate hosts, they are considered as one of the most important vectors of zoonotic pathogens. Ticks can transmit a wide variety of bacteria, parasites and viruses^1–3^. In human and veterinary medicine, most tick-borne pathogens are transmitted by various hard ticks belonging to genera *Ixodes, Haemaphysalis, Dermacentor, Hyalomma* and *Rhipicephalus* and by certain soft ticks belonging to genera *Argas* and *Ornithodoros.* Among tick-borne pathogens, 170 tick-borne viruses (TBVs) were identified and belong to nine virus families and twelve virus genera. Viruses transmitted via tick bites can cause various symptoms in humans and animals, ranging from mild febrile illness to neurological disorders or even haemorrhagic fevers^4,5^.

The oversite and existing gaps in our knowledge of ticks and TBV are partly due to the difficulty of setting effective experimental models to assess vector-competence or study virus-tick interactions in general. In fact, establishing and maintaining laboratory tick colonies and performing experimental infections of ticks remain a challenge^6^. The difficulties relate to the obligate hematophagous nature of hard ticks. Therefore, they need an animal host to feed on and reproduce, requiring lengthy feeding times and ingesting larger blood meals than any other known hematophagous arthropod. Tick-borne viruses received much less attention than viruses transmitted by mosquitoes, especially in terms of interaction with their arthropod vectors. Competent ticks acquire a given arbovirus during their feeding on infected vertebrate hosts or during co-feeding with infected ticks. The virus is then transmitted during the next blood meal after an extrinsic incubation period over which the virus will replicate and disseminate throughout the tick body. Hard ticks take only one blood meal per stage (larva, nymph and adult), meaning that the trans-stadial transmission of the virus is necessary for transmission to a new vertebrate host upon the following blood meal. Indeed, TBVs must survive changes occurring during moulting and maintain their infectivity into the following developmental stage^7^. Because transmission of viruses occurs when saliva is injected during feeding upon the host, the virus must reach and infect the tick salivary glands^8^. The complexity of the whole process starting with virus acquisition and progressing to infection of a new host, involves delicate molecular interactions between viruses and their vectors. Investigating these interactions facilitates the identification of elements and pathways used/abused by TBVs, which could be targeted to attempt blocking virus replication and control TBV-related diseases^9^. Therefore, reproducing transmission cycles of tick-borne viruses under controlled laboratory conditions remains an important prerequisite to study their interactions with respective vectors.

In order to study the complex of ticks and the pathogens they transmit, laboratory tick infection methodologies have been developed^6^. Several methodologies are in use in laboratories such as feeding on a live animal, capillary feeding, inoculation or immersion. Many of these techniques were used to infect ticks with bacteria and/or parasites. For viruses the most widely used methods are intrathoracic inoculation (bypasses the gut barrier), anal pore microinjection (does not bypass gut barrier) or feeding on a vireamic host^10–13^. The main aim of our study is to adapt/develop efficient tick infection methodologies, including an artificial feeding system (AFS)^14^ or an immersion technique (IT)^15^. Developing infection methods which allow selective infection of ticks with TBVs facilitates the study of pathogens of interest in the arthropod vector. The AFS relies on ticks feeding through an animal skin and has been validated as a model to infect ticks with *Babesia divergens*^14^, *Babesia venatorum*^16^, *Bartonella henselae*^17–19^ and *Bartonella birtlesii*^20^. The IT was validated to infect *Ixodes scapularis* with *Borrelia burgdorferi*^15^ and Langat virus (LGTV)^21^. Ticks were directly immersed into a suspension of the pathogen and further fed on a vertebrate host immediately thereafter. Tick infection models with tick-borne encephalitis virus (TBEV, genus *Flavivirus*) have been previously established and validated under laboratory settings. The main techniques include parenteral inoculation and co-feeding on mice^22–24^.

*Ixodes ricinus* is the primary vector of a wide range of pathogens and the most abundant and widely distributed tick species in Europe^25^. In our study, we used viruses belonging to genera *Flavivirus* or *Orbivirus* to infect *I. ricinus*. TBEV is known to be transmitted by *I. ricinus* and responsible for severe neurological illness of humans in Europe and Asia^26^. It was used as a positive control to assess the efficacy of both AFS and IT as infection methods. Kemerovo virus (KEMV, genus *Orbivirus*) is suspected to be the causative agent of encephalitis cases in humans in central Europe and Russia^27,28^. AFS and IT were used to assess vector competence of *I. ricinus* for KEMV. The virus has been isolated/detected in *I. persulcatus* and *I. ricinus*^29^. Up until this study, assessing vector competence of *I. ricinus* for KEMV has never been attempted. Assessing the efficacy of both infection techniques was based on the three criteria of vector competence: (i) virus acquisition by ticks, (ii) trans-stadial transmission, and (iii) transmission of the viruses to a vertebrate host.

## Results

### Artificial feeding and immersion techniques as tools to assess vector competence of *I. ricinus* for TBEV

#### TBEV acquisition by *I. ricinus* ticks and trans-stadial transmission

The feeding of larvae on clean or TBEV-spiked blood through membrane skin lasted for ten days. Feeding behaviour was not affected by the addition of virus into the sheep blood (*p* value = 0.18) (Table 1). Larvae which were immersed in the TBEV suspension fed for only 4 days on mice and the calculated percentage of engorgement is higher compared to membrane skin feeding (*p* < 0.001) (Table 1). Using the AFS, 70% of engorged larvae and 75% of nymphs were found positive for TBEV RNA (Table 1). After immersion in TBEV suspension, 72% of engorged larvae and 48% of nymphs were positive for TBEV RNA. The infection rate of larvae with TBEV was not impacted whether the method was AFS or IT (*p* = 0.88). However, the success of trans-stadial transmission of TBEV from engorged larvae to moulted nymphs was higher using the AFS than the IT (*p* < 0.001) (Table 1). Moreover, after feeding of immersed larvae, two mice showed clinical signs of infection and died on days 9 and 11 post-infestation. The last mouse which also showed clinical signs of infection, recovered and survived until the end of the experiment. Though the presence of TBEV RNA was not tested in these mice, we suspect some form of non-biological transmission of the virus by the larvae. Indeed this could be a mechanical transmission as suggested for lumpy skin disease transmission by *Aedes aegypti*^30^ or transmission of specific plant arboviruses by aphids^31^, is a matter which will require further investigation.

**Table 1:** Engorgement rates of larvae and TBEV infection in engorged larvae and nymphs infected at larval stage after artificial feeding (AFS) or immersion techniques (khi-2 test, alpha = 5%, *: *p* value < 0.001).

Tested parameters	Control ticks fed on clean blood using AFS	Ticks fed on TBEV-spiked blood using AFS	Ticks immersed in TBEV suspension followed by feeding on CD1 mice
% of engorged larvae	23.16% (2085/9000)	22.32% (2009/9000)	78.32%* (224/286)
% of infected larvae	0% 0/20	70% (14/20)	72% (18/25)
% of infected nymphs	0% 0/20	75% (15/20)	48%* (39/82)

#### Transmission of TBEV to a vertebrate host

The ability of nymphs infected at the larval stage to transmit TBEV to a vertebrate host was assessed by feeding on mice. Twelve months post moulting, nymphs moulted from larvae fed using AFS were placed on one IFNAR^−/−^ mouse. After feeding, real-time RT-PCR was used to detect TBEV in engorged nymphs and mouse samples. The engorged nymphs were tested positive for RNA TBEV. IFNAR^−/−^ mouse died six days post-infestation, and the virus was detected in the brain, spleen, liver, heart and lungs with Ct values ranging from 20.34 to 24.25.

Nymphs which moulted from larvae immersed in TBEV suspension were left for 3–12 months before being fed on 14 CD1 mice. After feeding, TBEV was detected in 60.0% of engorged nymphs (15/25). Out of the 14 mice, twelve (86%) died or showed typical symptoms of TBEV and were hence euthanised at the humane endpoint of the disease. The mean survival time was 11.6 days (± 1.75) after infestation. The remaining two mice, which had survived, presented symptoms of TBEV but recovered ultimately.

### Assessing vector competence of *Ixodes ricinus* ticks for KEMV using artificial feeding or immersion techniques

#### KEMV acquisition by ticks and trans-stadial transmission

Larvae feeding on membrane skin lasted seven days in total. The first engorged larvae were collected on day 5. A total of 1120 larvae were collected from the AFS containing the clean sheep blood and another 581 larvae were collected from the AFS containing the KEMV-spiked blood. The presence of KEMV in the AFS seems to reduce the feeding success of larvae (*p* < 0.001) (Table 2). Engorgement of larvae immersed in KEMV suspension was more efficient than engorgement using the AFS (*p* < 0.001) (Table 2). The presence of KEMV in engorged larvae was confirmed by testing randomly 30 individuals immediately after feeding using AFS. About 83.3% of tested larvae were positive for KEMV RNA. Engorged larvae from the control setting (non-spiked blood) were consistently found negative for KEMV by RT-PCR. The mean infection rates of engorged larvae after immersion was 26% (ranged from 0 to 60%, according to the tested batch), revealing a significant difference between the two infection methods (*p* < 0.001, Table 2). No clinical signs or mortality were observed in mice after feeding of immersed larvae. Mouse blood remained negative for KEMV RNA up until the end of feeding five days post-infestation.

**Table 2:** Engorgement rates of larvae and KEMV infection in engorged larvae and nymphs infected at larval stage after artificial feeding (AFS) or immersion techniques (khi-2 test, alpha = 5%, *: *p* value < 0.001).

Tested parameters	Control ticks fed on clean blood using AFS	Ticks fed on KEMV-spiked blood using AFS	Ticks immersed in KEMV suspension followed by feeding on BALB/C mice
% of engorged larvae	56% (1120/2000)	29%* (581/2000)	73% (886/1200)
% of infected larvae	0% 0/30	83.3% 25/30	26,25% (21/80)*
% of infected nymphs	0% 0/10	40% 4/10	0* 0/25

The trans-stadial transmission from larvae to nymphs was assessed, immediately after the moulting using real-time RT-PCR by testing 10 and 25 nymphs after AFS or IT, respectively. A significant difference was observed between the two infection techniques with infection rates in nymphs of 40% using AFS, and 0% with the IT (*p* < 0.001, Table 2). Five and seven months after larval feeding, an additional 25 unfed nymphs which moulted from larvae fed using AFS, were pooled and all tested negative by real-time PCR for the presence KEMV RNA.

#### *I. ricinus* nymphs infected at the larval stage fail to transmit KEMV to a vertebrate host

Five to seven months post-moulting, nymphs were fed on 4 BALB/c and 4 IFNAR^−/−^ mice for nymphs from immersed larvae and 4 BALB/c and 4 IFNAR^−/−^ mice for nymphs from AFS larvae. Feeding on mice lasted for 6 days. The mice were observed for 10 days post-infestation until the end of the experiment. Blood samples were collected on days 0, 5 and 10 to follow up the viraemia. On day 10 post-infestation, mice were euthanised and organs (brain, liver, spleen, lungs, heart and kidneys) were collected. Engorged nymphs, mouse organs and blood samples were tested by real-time RT-PCR for KEMV RNA. All samples were tested negative ruling out the possibility of a KEMV transmission to the mice by the feeding ticks.

### Comparison of TBEV and KEMV acquisition by ticks and trans-stadial transmission

**Figure 1:**
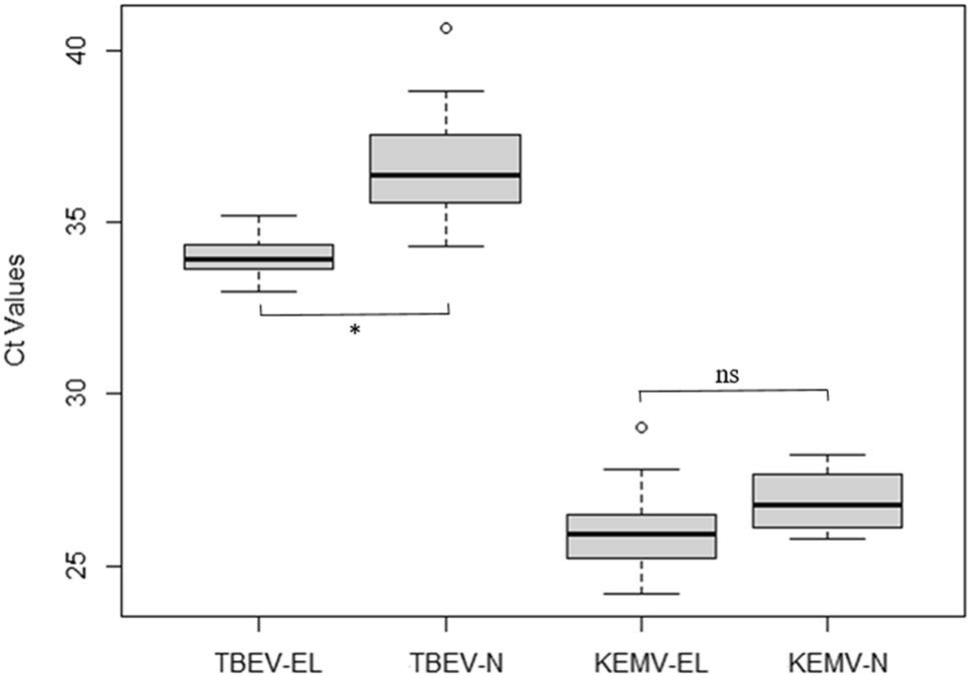
Detection of KEMV Seg-2 and TBEV envelope genes by real-time RT-PCR in lysates of individual *Ixodes ricinus* after infection using the artificial feeding system—EL: Engorged Larvae, N: nymphs (N infected at the larval stage) and comparison of mean Ct values for each group (Kruskal–Wallis test, alpha = 5%, *: *p* value < 0.001). The lower and upper lines represent the lower (Q1) and upper (Q3) quartiles. The median is represented in the diagram and data falling outside the Q1-Q3 range are plotted as outliers of the data (circle).

We used TBEV experiments as positive controls for the feasibility of artificial infections with KEMV. As shown in Tables 1 and 2, TBEV and KEMV infection rates of larvae with the AFS were similar (*p* = 0.26). Trans-stadial transmission tended to be much better with TBEV than with KEMV (*p* = 0.06). The presence of KEMV or TBEV genomes in the ticks correlates with the uptake of viral particles by the larvae during artificial feeding or immersion. Acquisition using the IT was more efficient with TBEV than with KEMV (*p* < 0.001). Trans-stadial transmission of KEMV was not confirmed using this technique. The distribution of Ct values of engorged larvae and moulted nymphs is shown in Fig. 1 after tick infection with TBEV and KEMV using AFS. Because KEMV cDNA was pre-amplified, the real-time RT-PCR signals for KEMV are earlier than those for TBEV (Fig. 1). The levels of TBEV RNA are lower in nymphs after moulting in comparison with engorged larvae (*p* < 0.001). Levels of KEMV RNA appear to be similar in engorged larvae and nymphs after moulting (*p* > 0.05).

## Discussion

This study investigated two laboratory tick infection methods for tick-borne viruses, namely an AFS^14^ and an IT^21^. *I. ricinus* is known to be a competent vector for TBEV^32^. This virus as a positive control to study the potential of the two infection methods for testing tick vector competence. We also assessed vector competence of *I. ricinus* for KEMV, a tick-borne zoonotic orbivirus. We have shown that TBEV can successfully infect *I. ricinus* using either methods and was efficiently passed from the infected ticks to a susceptible mouse model, as confirmed by the development of specific clinical signs and the detection of TBEV RNA in the mouse blood and organs. Our results also shown that the AFS is a more efficient method to infect ticks with TBEV than the IT. In addition, ticks were efficiently infected with KEMV using the AFS, while the IT failed to achieve infection. However, no KEMV transmission from infected ticks to mice as a model vertebrate host was observed.

Both infection methods used in this study, have their advantages and limitations. These techniques allow experimental tick infection without the requirement for viraemic animals They are also safe methods which avoid the use of glass needles required to inject a virus suspension into ticks^10,11^. Though a versatile technique, intrathoracic injection bypasses the gut barrier and hence is far from representing a natural route of infection^6,10–12^. Oral feeding remains the gold standard to assess key elements of virus-ticks interaction, especially considering that the gut barrier is an important aspect of vector competence. For instance, Dhori and Dugbe viruses are two tick-borne viruses transmitted by *Hyalomma dromedarii/H. marginatum* or *Amblyomma variegatum*. Both viruses replicate efficiently in *R. appendiculatus* haemocoel and are transmitted trans-stadialy and to a vertebrate host when the virus is injected into the tick. However, this tick species is not a competent vector of these two viruses and no replication, trans-stadial transmission nor transmission to a vertebrate host were observed when ticks were orally infected using virus spiked blood^33^. However, for the immersion method, it is not clear whether the virus is taken up by oral absorption or diffuses into the tick body through various pores. This method has also the disadvantage of requiring live animals to feed ticks which have been immersed into the virus suspension. Nevertheless it is a low cost method which is easier to set up and is less time consuming than the AFS. For larvae, the IT lasts 4 days, which spans the immersion of the ticks and the feeding on mice. Yet, the major limitation of IT is the generation of cohorts of infected ticks with an equal pathogen burden. In this study, the number of infected ticks with either virus using the IT was lower than using AFS. For TBEV infection, we obtained 224 engorged ticks after immersion, with an estimated infection rate at 72%, whereas using AFS we obtained 2009 engorged ticks with a comparable infection rate. Comparatively, the AFS lasts a minimum of 7 days with daily blood changes. It is also noted that our results with TBEV or KEMV suggest that the efficacy of the immersion method could rely on the nature of the virus/virus family. Unlike TBEV, we did not observe trans-stadial transmission of KEMV from larvae to nymphs using IT, when such a transmission was demonstrated with AFS.

Using the IT, the number of obtained engorged ticks is limited for further analysis due to a limited number of ticks per animal and limited number of animals used for ethical reasons. By contrast, the AFS allows feeding of a higher number of ticks^6^. AFS is a better method in terms of infection with TBEV and KEMV and it generates a larger cohort of infected ticks. In addition, it is an infection technique that is closer to the natural mode of tick infection (feeding on an infected viraemic vertebrate). However, it appears that the success of tick feeding is higher with live animals than upon using AFS. In addition, and despite good engorgement rates for control ticks in the KEMV experiment (56%), the observed engorgement rates with blood spiked with KEMV (29%) or with TBEV (22 to 23% ) were lower than those described in the scientific literature (ranging from 43 to 84.5%).^17–19^ Indeed, one of the limiting factor with this method is the unpredictability of feeding rates due to tick behaviour, as ticks may resist to feed, but this can also be a problem with feeding on live animals^6^.

The IT was efficient with TBEV, as observed with significant infection rates in nymphs which moulted from immersed larvae (48%). This technique was successful with LGTV in *I. scapularis* ticks with similar infection rates in engorged larvae of 96% and 87% in nymphs after moulting^21^. However, it was not the case with KEMV where the infection rates of larvae and nymphs after moulting were 26.25% and 0%, respectively. One possible explanation of the observed differences in infection efficiency can be the virus titre in the suspension used for immersion of larvae. In our study, the TBEV titre (2.5 × 10^8^ PFU/mL) was 100-fold higher than KEMV titre (4.4 × 10^6^ PFU/mL). In the LGTV study, the virus titre was 10^7^ PFU/mL^21^. The nature of the virus may also provide additional explanations to the observed differences. TBEV and LGTV are both enveloped flaviviruses with a single-stranded RNA genome, while KEMV is an orbivirus having a dsRNA genome and is non-enveloped. Thus, the two groups of viruses differ substantially in terms of structure of the virus particles (enveloped versus non-enveloped) and particular mechanisms involved in cell entry (endocytosis versus fusion) and tissue dissemination^34–39^. Furthermore, during the feeding on a vertebrate host, ticks inject saliva that modifies the local environment at the bite location. It has been demonstrated that saliva plays a role in the transmission of tick-borne pathogens to a vertebrate host and also in the pathogens acquisition^40^. Certain molecules present in vector saliva substantially influence infectivity and survival of TBVs in the tick^8^, and it has been shown that salivary proteases enhance infectivity of insect-borne orbiviruses^38^. If we assume that KEMV was taken up through body pores other than the mouth parts, its infectivity may have been less efficient to the larval tissues. Consequently, as the IT is further from a natural mode of feeding than AFS, it might influence infection efficacy of specific viruses and AFS remains a better tool to study the virus-tick interactions particularly to evaluate vector competence.

Like TBEV, KEMV was associated with *I. ricinus* in the field^29^. KEMV was isolated from these ticks in 1964 in Czechoslovakia^41^ and Vologda region in Russia in 1975^29^. However, up until this study, vector competence studies of *I. ricinus* for KEMV has been never attempted. Our results cast reasonable doubt about *I. ricinus* being a natural vector for KEMV. We have shown that *I. ricinus* larvae can be infected with KEMV during a blood meal and remain infected after moulting (two weeks after) when infected using our AFS. Although a relatively high infection rate was observed in engorged larvae (83.3%), a low efficacy of trans-stadial transmission resulted in only 40% of infected nymphs two weeks after moulting was reported. The first trials of KEMV transmission were performed five months post-moulting and we did not observe transmission to mice. The virus seemed to have disappear from the nymphs which were found negative for KEMV RNA 5 months post-moulting. These results suggest that the ticks cleared the virus during this period. Arthropods have antiviral innate immune responses. The most robust antiviral response is RNA interference (RNAi) which targets viral RNA degradation, thus keeping virus replication in check^42^. This pathway is well known in mosquitoes but not clearly understood in ticks. A previously published study showed the involvement of Dcr and Ago proteins in the antiviral RNAi response after TBEV or LGTV infection of IDE8 tick cells (a cell line derived from *I. scapularis*)^43^. It is possible that *I. ricinus* antiviral immunity cleared the virus or even KEMV failed to disseminate from the site of entry into the tick to other tick organs, a process that is crucial to infect salivary glands and onward transmission to vertebrate hosts. Our studies showed that *I. ricinus* larvae were infected with the virus, which survived moulting into nymphs, but real-time RT-PCR detecting KEMV RNA was performed with RNA extract from whole ticks, and localisation of the virus within the nymphs has not be identified so far.

The detection of KEMV RNA in larvae extract after immersion may have resulted from particles which remained on the body surface or from particles which have been taken up by the tick though body pores without further replication. Larvae moulting into nymphs is accompanied by a loss of the cuticle (apolysis)^44^ and the loss of any potentially virus particles present on the surface of their bodies. However, it should be noted that the non-biological transmission by immersed larvae via a possible contamination of the mouthparts suspected for TBEV, was not observed with KEMV.

Moreover, interaction between arboviruses and their vector is a complex process. Vector competence is genetically determined and could rely on a particular virus strain and on a particular arthropod geographical strain. This has been shown previously for other arboviruses such as TBEV, bluetongue, Rift Valley fever virus or chikungunya virus^45–50^. The *I. ricinus* ticks we used in our studies were from Slovakia. The prevalent serotypes of KEMV in Slovakia are Lipovnik (LIPV) and Tribeč (TRBV) virus as previously reported^41^, and both viruses has been isolated from *I. ricinus*. The KEMV strain used in this study was isolated in Russia in 1962 where several KEMV isolates were obtained from *I. persulcatus*^27^, the principal *Ixodid* tick in that geographical region^51^. Thus the geographical origin of the tick population could be a contributing factor to the observed results. We have currently ongoing studies with laboratory reared *I. persulcatus* larvae from Russia, which will provide clear cut information about the role of this tick species in maintaining KEMV in the field and evaluation of our experimental infection methods. Studies with *I. ricinus* from Russia would also provide additional valuable information as to whether geographical variants of this tick may influence competence. Alternatively, an assessment of vector competence of *I. ricinus* from Slovakia towards TRBV or LIPV could also shed light on potential genetic traits that are virus and tick population related.

## Conclusion

We have assessed two experimental techniques to infect ticks with tick-borne viruses. We validated two tick infection methods, an AFS and an IT, with TBEV using *I. ricinus* and showed vector competence of *I. ricinus* for TBEV in the laboratory*.* Our preliminary results suggest that *I. ricinus* appears to be poorly competent for KEMV but further studies are required to confirm such a result. Both techniques presented in this study have their advantages and limitations but as the IT is further from a natural mode of feeding than AFS, it might influence infection efficacy of specific viruses and AFS remains a better tool to study the virus-tick interactions, tick co-infections (viruses, bacteria and parasites) and more particularly to evaluate vector competence. These studies are essential to understand mechanisms of evolution, emergence/re-emergence of tick-borne pathogens, and to identify new methods to control tick-borne diseases for which an urgent public health response is needed.

## Methods

### Experimental design

**Figure 2:**
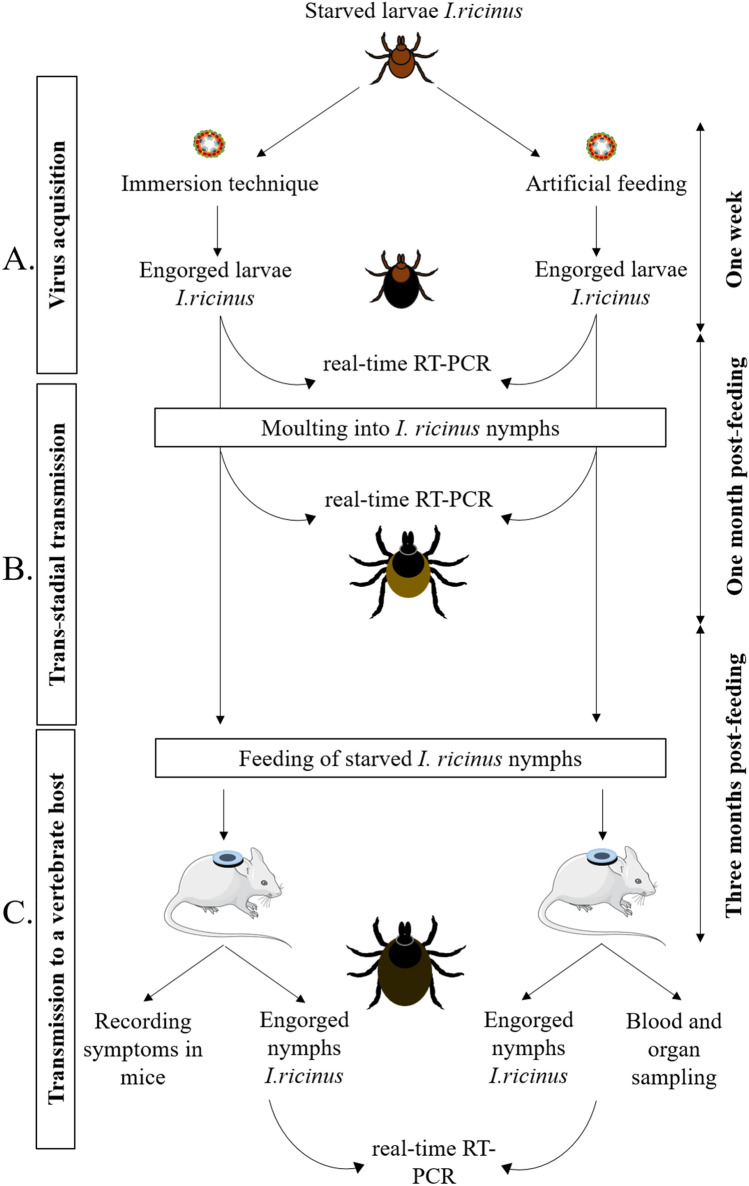
Schematic representation of the experimental design for assessing vector competence of ticks for pathogens through immersion technique and artificial feeding. (**A**) pathogen acquisition, (**B**) trans-stadial pathogen transmission and (**C**) transmission of the pathogen to a vertebrate host. Ticks were allowed to feed to repletion and harvested immediately after feeding. Real-time RT-PCR is performed to specifically detect the pathogen’s RNA in ticks and mice at different steps of the protocol.

The experimental design of the study is shown in Fig. 2. It involves virus acquisition by *I. ricinus* larvae (Fig. 2A). After feeding, engorged larvae need approximately one month to moult into the nymphal stage. The trans-stadial transmission of the virus was assessed immediately after the engorged larvae have moulted into nymphs. A real-time RT-PCR assay was used to quantify virus replication (Fig. 2B). Nymphs were placed on mice for at least two months and up to one-year post-moulting to assess virus transmission to a vertebrate host (Fig. 2C).

### Viruses

#### Tick-borne encephalitis virus (TBEV)

Ticks were infected with TBEV strain Hypr. This strain was originally isolated in 1953 in the Czech Republic from the blood of a child with TBE^52^. TBEV Hypr was passaged 4 times in suckling mice brains and twice in Vero E6 cells^53^ and was used in the experiments.

#### Kemerovo virus (KEMV)

A KEMV virus stock was prepared in BSR cells (a clone of BHK-21^54^). The virus was originally isolated from the brain of a patient who died of acute encephalitis in 1962^27^. This isolate was further amplified once in suckling mouse brain and passaged twice in Vero cells in 1969. Before use, the initial stock of the virus was propagated three times in BSR cells.

### Plaque assay

TBEV titre was determined using plaque assay using either porcine PS or monkey kidney Vero E6 cells. On the day before the assay, cells were plated (1.5 × 10^5^ cells/well) in 24-well plates with Dulbecco's Modified Eagle Medium (DMEM) supplemented with penicillin (50 IU/mL), streptomycin (50 µg/mL) and 10% foetal bovin serum (FBS) at 37 °C under 5% CO_2_. Ten-fold serial dilutions of TBEV were prepared in serum-free DMEM. Two hundred microliters of each dilution were added per well for 1.5 h at 37 °C. Cells were covered with a carboxy-methyl cellulose (CMC) overlay in DMEM. After five days of incubation at 37 °C, cells were rinsed three times with phosphate-buffered saline (PBS) and fixed/stained with a 10% formaldehyde solution containing 0.1% crystal violet. TBEV titre was determined as 2.5 × 10^8^ PFU/mL.

KEMV titre was also determined by plaque assay as described previously^55^. On day 5 post-infection, BSR cell debris were pelleted by centrifugation at 2000 g for 10 min at 4 °C. The supernatant was discarded, and cell debris were suspended in 5 mL of serum-free culture medium and treated with an equal volume organic solvent, Vertrel XF (Sigma-Aldrich, United States), to free virus particles from cell debris as described previously^56^. The mixture was shaken manually and centrifuged at 2000 g for 5 min. The aqueous phase, containing free virus particles, was carefully recovered. Briefly, BSR cells were plated (1 × 10^5^ cells/well) in 24-well plates a day before running the assay in DMEM supplemented with penicillin (50 IU/mL) and streptomycin (50 µg/mL), 10% of FBS at 37 °C, 5% CO_2_. Ten-fold serial dilutions of the virus were prepared in serum-free DMEM and 250 µL of each dilution was added per well for 2 h at 37 °C. Cells were then covered with 1 mL of molten 1% low melting point agarose in DMEM. After incubation at 37 °C for 5 days, 2 mL of 10% formaldehyde was added to each well. Cells were stained with 0.1% naphthalene-black solution after removal of the agarose plugs, and plaques counted. The virus titre was determined as 4.4 × 10^6^ PFU/mL.

### Ticks

For TBEV infection studies, we used *I. ricinus* larvae derived from the 1^st^ generation of ticks collected in the Murbach forest from the Alsace region in France, or from a laboratory colony of ticks initially collected in the Czech Republic. For KEMV infection studies, we used larvae from the 4^th^ generation of a laboratory colony provided by the Institute of Zoology, Slovak Academy of Sciences, Bratislava, Slovakia. Ticks were maintained at 22 °C with 95% relative humidity and a 12 h light/dark cycle in the animal facilities as described in^14^. In each case, after egg-laying, females were tested for KEMV and TBEV RNA before larvae were used in the experiments.

### Mice

TBEV-infected ticks from immersion experiments were fed on three weeks old specific pathogen-free (SPF) female mice of the ICR (CD1) strain (Charles River Laboratories, Sulzfeld, Germany) known for their susceptibility to TBEV infections^57^. TBEV-infected ticks from the artificial feeding experiments, were fed on adult (4–5 weeks old) type I interferon receptor knock-out mice (IFNAR^−/−^, genetic background: A129SvEvBrd)) kindly provided by Prof. Michel Aguet (Ecole Polytechnique Fédérale de Lausanne)^58,59^. These mice are also susceptible for TBEV infections and develop severe clinical signs ending by death around day 6 post-infection^60^.

BALB/c female mice (7 weeks old) (Charles River Company, Saint Germain Nuelles, France) and IFNAR^−/−^ female mice (10 weeks old) were used for feeding KEMV-infected ticks. BALB/c mice were used for feeding after immersing ticks in the viral suspension and for assessing the transmission of the virus to the vertebrate host. IFNAR^−/−^ mice were used for assessing the virus transmission from the infected ticks to the host only. The use of these strains is warranted by previous findings. BALB/c mice are commonly used for infection with orbiviruses^61^, and IFNAR^−/−^ usually develop severe clinical signs of infection with KEMV, including neurological signs, haemorrhage, and prostration (Camille Migné PhD thesis, in progress). In addition, IFNAR^−/−^ mice are well known to be susceptible for orbivirus infections^55,62^.

The mice were individually held in plastic cages with wood-chip bedding, fed ad libitum and kept in standardised conditions (22 °C, 65% relative air humidity, 12:12 light/dark cycle) at the SPF animal facilities of the Institute of Parasitology and ANSES**.** The present research was conducted in compliance with all relevant European Union guidelines for work with animals and with the Czech or French national law guidelines on the use of experimental animals and protection of animals against cruelty. Experimental protocols were approved by the Committee on the Ethics of Animal Experimentation at the Institute of Parasitology, the Departmental Expert Committee for Approval of Projects of Experiments on Animals at the Czech Academy of Sciences (Permit No. 29/2016) and by ANSES-ENVA-UPEC Ethics Committee for Animal Experimentation (Agreement Number: 13-021, 19-004, 19-005 and 19-028).

### Tick infection by artificial feeding

**Figure 3:**
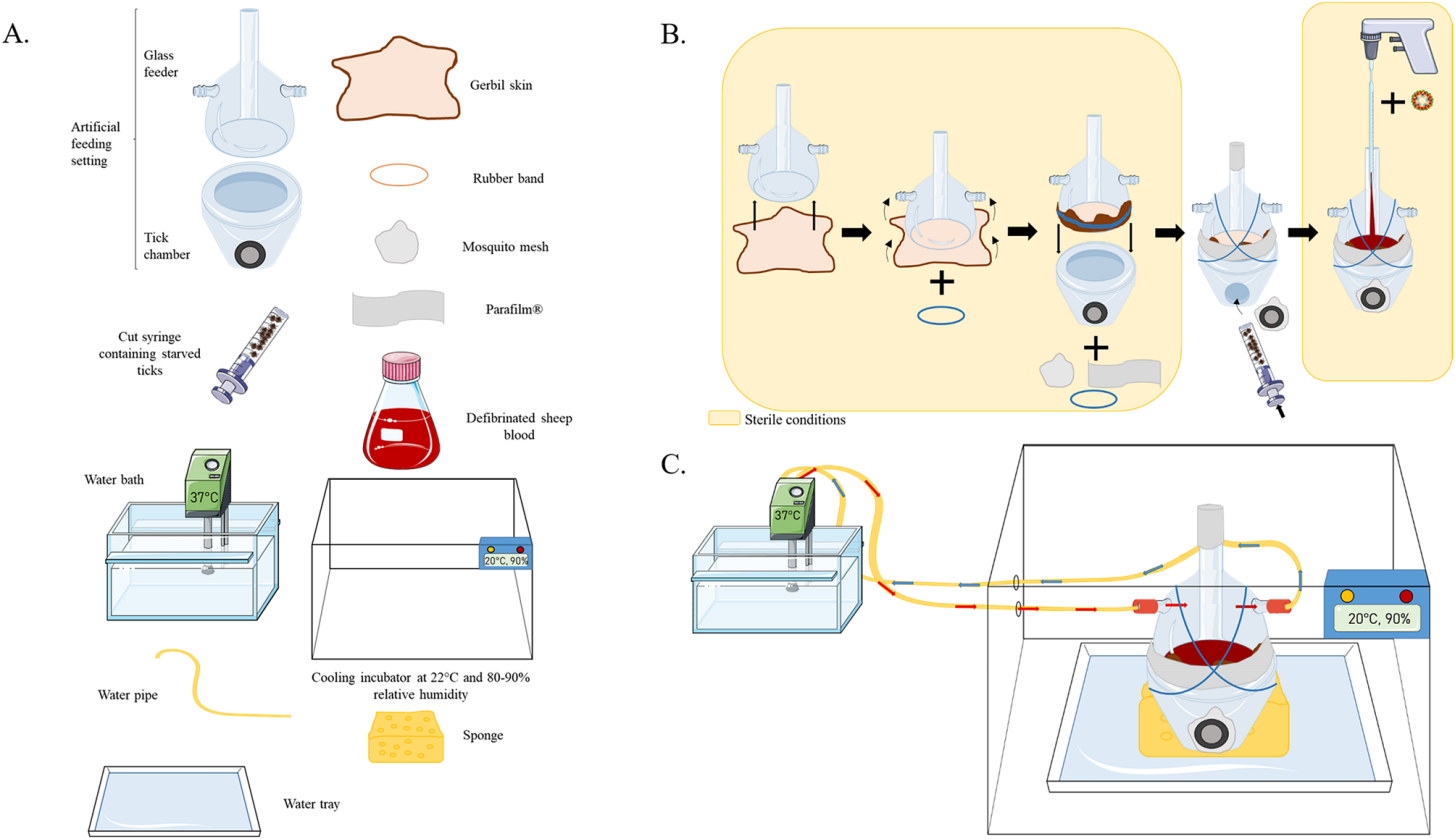
Schematic representation of (**A**) the necessary material, (**B**) the setting of the tick artificial feeding system and (**C**) the complete assembly of the system (adapted from^13^).

The previously described tick AFS^14^ was used to infect *I. ricinus* with TBEV and KEMV. The system, material and the setting are shown in Fig. 3. All procedures with the artificial feeding system were performed under sterile conditions. A cut gerbil skin were used to feed larvae as described in^14^. Commercial defibrinated sheep blood was purchased from Eurobio scientific (France). Before use, the blood was supplemented with 17 U/mL of heparin (Merck, Germany). Gentamicin (Invitrogen, Germany) and Amphotericin B (Invitrogen, Germany) at a final concentration of 10 mg/mL and 250 µg/mL, respectively. For infection studies, the blood was directly spiked with 10^5^ PFU/mL for TBEV^63–65^ or 10^4^ PFU/mL for KEMV^55^. For each experiment, control and infected settings were used in parallel. Ticks were placed in the tick chamber and enclosed with a mosquito mesh allowing them to breathe. To attract ticks, the setting was maintained at 37 °C by a circulating warmed water circuit (Fig. 3C)^14^.

For TBEV infection, triplicate feeders (per experiment) were used for virus-spiked or clean bloods. Approximately 3,000 larvae were placed in each chamber. Ticks for KEMV experimentation were a gift from colleagues in Slovakia. We thus used a single setting for KEMV-spiked blood and a single control setting each containing approximately 2,000 larvae, availability of ticks being here a limiting factor.

#### Assessment of virus infectivity in sheep blood and frequency of blood changes

The frequency of blood changes in the AFS was decided based on an initial assessment of virus infectivity in sheep blood spiked with cell-culture derived TBEV or KEMV.

TBEV infectivity in sheep blood was assessed over a time course of 30 h. The blood was spiked with 10^5^ PFU/ml using a fresh TBEV suspension. Blood samples were recovered from the setting at 0, 4, 8, 10, 24 and 30 h and titrated by plaque assay as described above. TBEV was titrated at different time’s intervals after blood spiking. After 10 h post-infection, the titre decreased by about 1.5 log.

Similarly, sheep blood was spiked with 10^4^ PFU/ml of fresh KEMV suspension and blood samples were recovered at 0, 4, 8, 10 and 24 h post-spiking and assessed by real-time RT-PCR. We observed a slight drop in KEMV genome copy numbers after 12 h (see Supplementary Figure S1).

Blood was changed daily for control ticks. We decided to replace TBEV or KEMV spiked sheep blood in the AFS twice a day to avoid any potential reduction of infectious virus titres in the system.

#### Tick collection

Ticks were allowed to feed to repletion. Feeding experiments usually took six days with an attachment rate to the membrane skin of around 90% and a repletion rate of 80–90% of engorged larvae^14^.

A randomly chosen subset of engorged larvae (20 and 30 engorged larvae for TBEV and KEMV settings, respectively) was placed at − 80 °C immediately after collection to further detect viral RNA.

### Tick infection through immersion technique

The IT was initially developed to infect *Ixodes scapularis* with *Borrelia burgdorferi*^15^ and Langat virus^21^ and adapted here for TBEV and KEMV.

#### Tick immersion in the virus suspension

**Figure 4:**
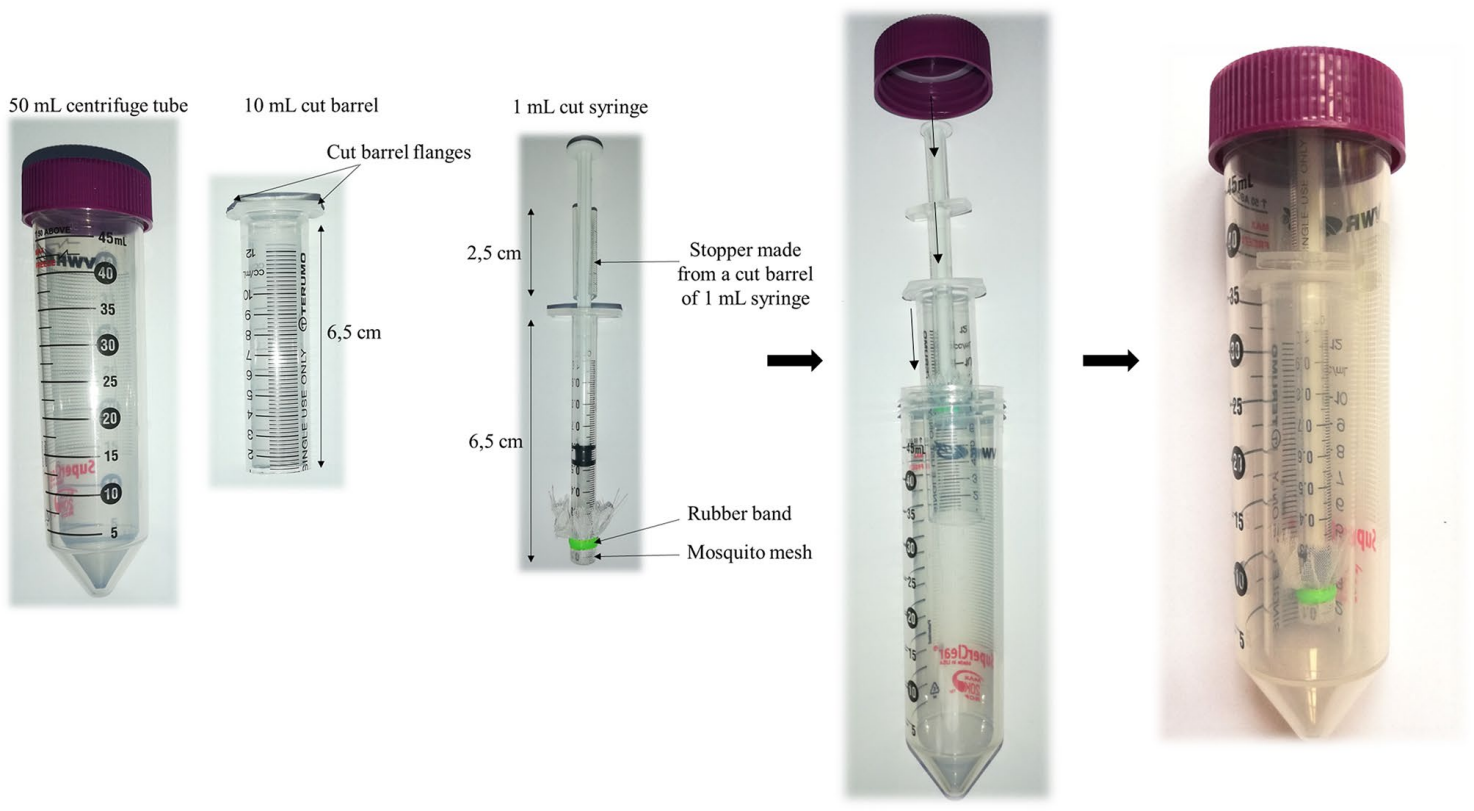
Material to infect ticks using the immersion technique.

**Figure 5:**
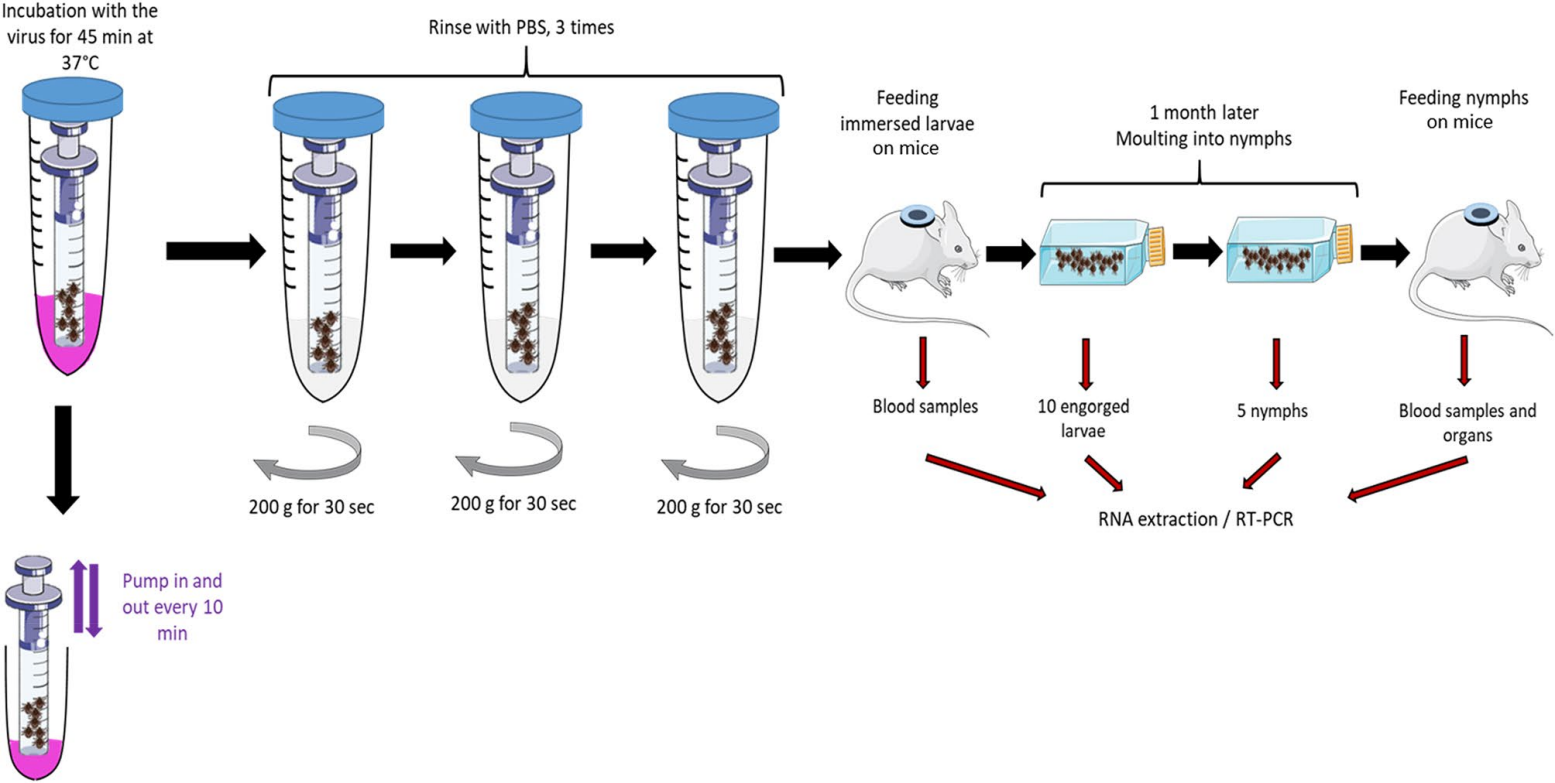
Schematic representation of the immersion technique used to infect ticks with KEMV and TBEV (adapted from^14,20^).

To immerse ticks easily, the tip of a 1 mL syringe was cut and covered with a mosquito mesh attached with a rubber band. Between 100 and 200 larvae were placed in this syringe, inserted into another syringe of 10 mL, the tip of which was also cut (acts as holder for the smaller syringe), and finally inserted into a 50 mL centrifuge tube (Fig. 4). Ticks were immersed in a non-diluted virus suspension (4.4 × 10^6^ PFU/mL for KEMV and 2.5 × 10^8^ PFU/mL for TBEV) of 5 mL for 45 min at 37 °C. The fluid was pumped in and out using the plunger every 10 min to ensure immersion of all ticks. Ticks were then rinsed three times with PBS and centrifuged at 200 g for 30 s to remove the supernatant between each washing (Fig. 5). The 50 mL falcon tube and the syringe holder were changed between the washings. Immediately after the procedure, ticks were placed on mice within a capsule system (feeding chamber) as described previously^66^. Briefly, feeding chambers were mounted on a shaved spot at the back of the mouse close to the neck. Mouse cages were placed over water reservoirs, which serve as traps to prevent escape of ticks in case of accidental chamber damage. Three female mice of the ICR (CD1) strain were used for feeding TBEV-immersed ticks and eight female BALB/c mice were used for feeding KEMV-immersed ticks.

#### Tick collection

Ticks were allowed to feed for four days. The first engorged larvae were collected on day three post-infestation and the latest on day four post-infestation. Symptoms of infection and/or mortality of mice were monitored throughout the experiments which lasted for 5 days post-infestation for KEMV and 11 days post-infestation for TBEV. Blood samples were collected only from mice used in the KEMV experiment. Engorged larvae were placed in flasks and maintained at a relative humidity of 95% at 22 °C. Between 9–12 engorged larvae per mouse were randomly chosen and stored at -80 °C to check for the presence of viral RNA.

### Virus transmission to mice following experimental infections

To check if nymphs which were infected at the preceding larval stage can transmit the virus to a vertebrate host, ticks were placed on mice within a capsule system as described above. Nymphs which moulted from larvae infected with TBEV were fed on IFNAR^−/−^ (larvae infected using artificial feeding) or CD1 (larvae immersed in TBEV suspension) mice. Nymphs which moulted from larvae infected with KEMV were fed on IFNAR^−/−^ mice or BALB/c mice. Symptoms of the infection and/or mortality were recorded for each mouse throughout the experiments. At the end of the experiments (6 and 10 days post-infestation for TBEV and KEMV, respectively), mouse blood and organs (brain, spleen, liver, heart and lungs) were sampled (except for TBEV/IT, where only symptoms and/or death were recorded). Presence of virus in mouse blood and tissue samples was assessed by real-time RT-PCR for TBEV and KEMV as described below. All procedures are summarised in Table 3.

**Table 3:** Experimental design of virus transmission from nymphs infected at the larval stage to a vertebrate host.

Methods used to infect larval stages	Virus	Infestation date after moulting (months)	Number of mice			Nymphs per mouse	Organ sampling	Blood sampling	End (days post-infestation)
			IFNAR^−/−^	BALB/c	CD1				
Artificial feeding	TBEV	12	1	–	–	2	Yes	Yes	6
	KEMV	5	4	4	–	30	Yes	Yes	10
Immersion	TBEV	3–12	–	–	14	10	No	No	30
	KEMV	5	4	4	–	30	Yes	Yes	10

### Virus detection by real-time RT-PCR

Virus acquisition by larvae, trans-stadial transmission from larvae to nymph, and transmission from nymphs to mice were assessed by detecting virus genome in both ticks and mouse organs and blood, using real time RT-PCR.

For RNA extraction, ticks were homogenised individually (except engorged nymphs after assessing TBEV transmission to the mice, which were pooled 2 by 2), in 350 µL of lysis buffer and 3.5 µL of β-mercaptoethanol with six stainless-steel beads (2.8 mm diameter) using Precellys®24 Dual homogeniser (Bertin, Paris, France) at 5500 × rpm for 20 s or a single sterile stainless-steel bead (5 mm diameter) in TissueLyzer II (Qiagen, Hilden, Germany) at 30 Hz for 3 min. RNA was then extracted from individual ticks, pooled ticks or 40 µL of whole mouse blood using NucleoSpin® RNA extract II (Macherey Nagel, Germany) as described by the manufacturer. All mouse organs were individually homogenised (½ heart, ½ lung, ½ spleen, ¼ liver, ½ brain) in 1 mL of DMEM culture medium supplemented with 10% FBS with six stainless-steel beads using the Precellys®24 Dual homogeniser at 5500 rpm for 20 s. RNA was extracted from 500 µL of homogenate using NucleoSpin® RNA extract II kit (Macherey Nagel, Germany). All RNA samples were eluted in 30 µL of RNase-free water and stored at − 80 °C until further used.

Tick or organ lysates were tested individually by real-time RT-PCR. Because the genome of KEMV consists of segmented double-stranded RNA, heat-denaturation is a prerequisite for a successful reverse transcription^67^. Thus, before real-time RT-PCR, the dsRNA is heat denatured at 99 °C for 5 min using an appropriate heating system, such as a thermal cycler. Renaturation of dsRNA must be prevented by immediately quenching the tubes on wet ice. Reverse transcription was performed using the qSript cDNA Supermix kit (Quanta Biosciences, Beverly, USA) as previously described^68^. The reaction was carried out in a final volume of 5 µL containing 1 µL of qScript cDNA Supermix 5X, 3 µL of RNase-free water and 1 µL of RNA. The thermal cycling programme consisted of a single cycle at 25 °C for 5 min, followed by one cycle at 42 °C for 30 min and one final cycle at 85 °C for 5 min. DNA preamplification was performed in presence of primers KEMV_F -GTCAGACGGATTTTCGACCTC- and KEMV_R -GCGAGCCAGATCCCGATGT, targeting genome segment 2 (encoding the VP2 protein). The preamplification was carried out in a final volume of 5 µL, containing 1 µL of 5X Perfecta Preamp Supermix (Fluidigm, USA), 1.25 µL of primers solution (0.2 µM final concentration each), 1.5 µL of distilled water and 1.25 µL of KEMV cDNA. The PCR cycling programme consisted of one cycle at 95 °C for 2 min, 14 successive cycles of denaturation at 95 °C for 15 s and amplification at 60 °C for 4 min. The pre-amplified DNA were 1:2 diluted and stored at − 20 °C until further used. The real-time PCR was performed using the LightCycler® 480 Probes Master kit according to the manufacturer's instructions (Roche Diagnostics, Germany). The following primers and probe were used: KEMV_F (final concentration: 0.5 µM), KEMV_R (final concentration: 0.5 µM) and KEMV_P ACGGGCCAACACTCGTTCATCACAG (final concentration: 0.25 µM )^68^. All samples were tested in duplicates. The PCR cycling programme consisted of one cycle at 95 °C for 30 s, 45 successive cycles at 95 °C for 10 s, 60 °C for 30 s and 72 °C for 1 s. The programme ended by a cooling cycle at 40 °C for 30 s.

TBEV RNA was detected using either the genesig Tick-borne encephalitis virus Polyprotein Advanced Kit (Primerdesign, UK) together with OasigTM lyophilised OneStep qRT-PCR Mastermix (Primerdesign, UK) or LightCycler® 480 RNA Master Hydrolysis Probes kit following manufacturer's instructions (Roche Diagnostics, Germany). The following primers and probes, targeting the 3’ non coding region (3’NCR), were used: TBEV_F GGGCGGTTCTTGTTCTCC (final concentration: 0.5 µM), TBEV_R ACACATCACCTCCTTGTCAGACT (final concentration: 0.5 µM) and TBEV_P TGAGCCACCATCACCCAGACACA (final concentration: 0.25 µM)^69^. The PCR cycling programme consisted of one cycle of reverse transcription at 63 °C for 3 min, followed by one cycle at 95 °C for 30 s, 45 successive cycles at 95 °C for 10 s, 60 °C for 30 s and 72 °C for 1 s. The programme ended by a cooling cycle at 40 °C for 30 s.

### Approval for animal experiments

The authors confirm the study was carried out in compliance with the ARRIVE guidelines.

## Supplementary Information

Supplementary Figure S1.

## Data Availability

Data are available upon request from the corresponding author.
