# Supplementary material for: Evaluation of two artificial infection methods of live ticks as tools for studying interactions between tick-borne viruses and their tick vectors
Source: Sci Rep. 2022 Jan 11;12:491. doi: 10.1038/s41598-021-04498-9 (PMC8752753; doi:10.1038/s41598-021-04498-9)
Supplement: Supplementary file 1 — Supplementary Figure S1. [file 41598_2021_4498_MOESM1_ESM.docx]

**Supplementary Material for**

**Evaluation of two artificial infection methods of live ticks as tools for studying interactions between tick-borne viruses and their tick vectors**

Camille Victoire Migné ^1,2*^, Vaclav Hönig ^3,4*^, Sarah I Bonnet ^1^, Martin Palus ^3,4^, Sabine Rakotobe ^1^, Clémence Galon ^1^, Aurélie Heckmann^1^, Eva Vyletova^3,5^, Elodie Devillers^1^, Houssam Attoui ^2^, Daniel Ruzek ^3,4^, Sara Moutailler ^1#^

^1^ Anses, INRAE, Ecole Nationale Vétérinaire d’Alfort, UMR BIPAR, Laboratoire de Santé Animale, Maisons-Alfort, F-94700, France;

^2^ UMR1161 Virologie INRAE, Anses, Ecole Nationale Vétérinaire d’Alfort, Maisons-Alfort, F-94700, France;

^3^ Institute of Parasitology, Biology Centre, Czech Academy of Sciences, Branisovska 31, 370 05, Ceske Budejovice, Czech Republic

^4^ Emerging Viral Diseases Research Group, Veterinary Research Institute, Hudcova 296/70, 621 00, Brno, Czech Republic

^5^ Faculty of Agriculture & Faculty of Science, University of South Bohemia, Branisovska 31, 370 05, Ceske Budejovice, Czech Republic

^*^ Authors contributed equally

^#^ Correspondence: Sara Moutailler, sara.moutailler@anses.fr

Supplementary Figure S1: Copy numbers of KEMV genome and virus titres (PFU/mL) of TBEV over 24 hours in sheep blood
